# Mechanisms and therapeutic opportunities of the ribotoxic stress response in cancer

**DOI:** 10.1002/1878-0261.70323

**Published:** 2026-09-02

**Authors:** Anastassiya Kim, Maryam Aslam, Elouise Torruella, Alphons Sony, Asif Rahman, Simira Smith, Columba de la Parra, Moira Sauane

**Affiliations:** ^1^ Department of Biological Sciences, Herbert H. Lehman College City University of New York Bronx NY USA; ^2^ Ph.D. Program in Biology, The Graduate Center City University of New York NY USA; ^3^ Department of Biological Sciences, Bronx Community College City University of New York Bronx NY USA; ^4^ Department of Chemistry, Herbert H. Lehman College City University of New York Bronx NY USA

**Keywords:** cancer, ribosome collision, ribosome stalling, ribotoxic stress response, stress‐activated protein kinase, ZAKα

## Abstract

Various environmental and endogenous stressors, including ultraviolet (UV) radiation, cytotoxins, and dysregulated translation, can induce ribosome stalling and collisions, disrupting protein homeostasis. The ribotoxic stress response (RSR) is a cellular surveillance mechanism that senses translational stress and activates stress signaling via the MAP3 kinase ZAKα and the stress‐activated protein kinases (SAPKs) p38 and JNK. This review outlines the molecular mechanisms behind RSR, distinguishes RSR from other translational stress response pathways, such as the well‐studied integrated stress response (ISR), discusses the role of RSR in key cellular processes, and presents new evidence linking RSR to cancer biology. We explore how ribotoxic stress is exploited by chemotherapeutic agents and other compounds to induce cancer cell death, and the potential limitations of such therapeutic strategy. Finally, we highlight future considerations for inducing the RSR pathway in cancer, highlighting both therapeutic potential and the challenges in this emerging field.

## Graphical abstract

Cancer cells' high translational demand creates opportunities to therapeutically target ribosome function. Ribosome stalling and collisions activate ZAKα and the ribotoxic stress response (RSR), which can trigger rapid, p53‐independent apoptosis in cancer. However, RSR can also promote cell‐cycle arrest, epithelial–mesenchymal transition, genome instability, and inflammation, highlighting both its therapeutic potential and possible risks in cancer.

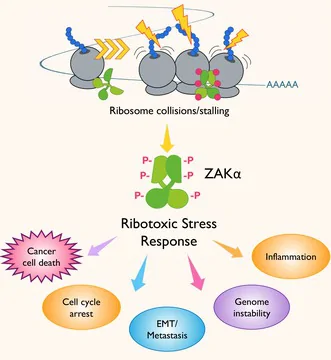

**Abbreviations**
ANS: anisomycin
APL: acute promyelocytic leukemia
ASCC: activating signal cointegrator 1 complex
ATF4: activating transcription factor 4
ATO: arsenic trioxide
CAR‐T: chimeric antigen receptor T‐cell
CDK: cyclin‐dependent kinase
DDR: DNA damage response
DHP‐2: (7‐[3‐fluoro‐4‐aminophenyl‐(4‐(2‐pyridin‐2‐yl‐5 6‐dihydro‐4H‐pyrrolo[1,2‐b]pyrazol‐3‐yl))]‐quinoline)
DOX: doxorubicin
DT: diphtheria toxin
eEF2: eukaryotic elongation factor 2
eIF2α: eukaryotic translation initiation factor 2 alpha
EMT: epithelial–mesenchymal transition
exoA: exotoxin A
GCN2: general control nonderepressible 2
HHT: homoharringtonine
ISR: integrated stress response
JNK: c‐Jun N‐terminal kinase
MAP3K: mitogen‐activated protein kinase kinase kinase
MAPK: mitogen‐activated protein kinase
METTL1: methyltransferase‐like 1
MHC‐I: major histocompatibility complex class I
MLK7: mixed lineage kinase 7
mTOR: mechanistic target of rapamycin
NK cell: natural killer cell
PI3K: phosphoinositide 3‐kinase
ROS: reactive oxygen species
RQC: ribosome‐associated quality control
RSR: ribotoxic stress response
SAM: sterile alpha motif
SAPK: stress‐activated protein kinase
SERBP1: SERPINE1 mRNA‐binding protein 1
TGF‐β: transforming growth factor beta
UV: ultraviolet
WGD: whole‐genome duplication
β‐TrCP1/2: beta‐transducin repeat‐containing protein 1/2

## Introduction

Cancer heterogeneity has been one of the toughest challenges in cancer therapeutics for several decades. Currently, cancer diagnostics, drug development, and basic research focus on targeted therapy and personalized approach. However, there are experimental limitations that curb discovery of new therapeutic targets and cancer biomarkers. Recent advancements in oncology, including CAR‐T cell therapy, gene therapy, oncolytic viruses, cancer peptide, and mRNA vaccines, are largely developed based on the genetic aberrations and transcriptomic data [1]. Yet, mRNA abundance accounts for only 40% of protein variability [2]. Hence, transcriptomic data do not accurately portray expression of functional proteins in a cell and is a limiting experimental approach in cancer research. Cancer adaptations and phenotypic plasticity are largely attributed to post‐transcriptional modifications and regulation of protein synthesis [1, 2].

Protein synthesis is a complex and well‐regulated cellular process. More importantly, cancer proliferation hinges on high protein synthesis capacity. Key oncogenic programs enable chronic proliferative signaling in cancer [3]. Aberrant oncogenic signaling pathways such as PI3K/mTOR, RAS/MAPK, and/or MYC converge on promoting translation initiation via the eIF4F complex and the elongation step via the elongation factor eEF2 [3]. Enhanced oncogenic signaling enables the high protein synthesis capacity of cancer cells, initiates translation of specific oncoproteins, and results in resistance to targeted therapy and enhanced cancer invasiveness [4, 5, 6, 7].

**Fig. 1:**
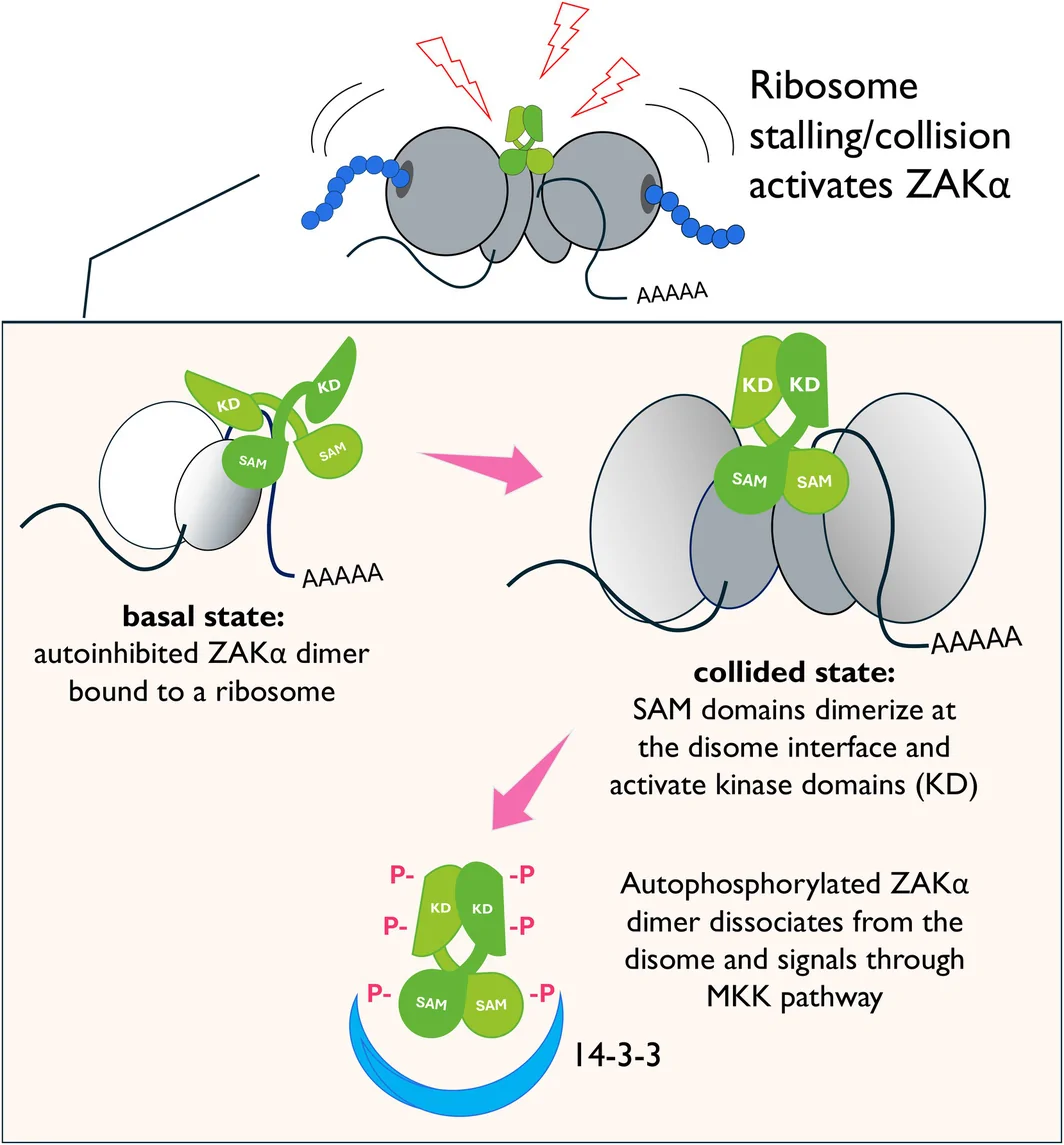
Model of ZAKα activation upon ribosomal collision. In the basal state, ZAKα dimer (green) binds to the 40s ribosomal subunit in autoinhibited conformation. Upon collisions, two ZAKα units are bound to two ribosomes at the interface of collision, which leads to SAM (sterile alpha motif) domain dimerization, stopping the inhibition of kinase domains (KD). Finally, ZAKα dimer becomes activated through autophosphorylation, detaches from the ribosomes, forms a complex with 14–3‐3 scaffold protein, and signals through the MKK (MAP kinase kinase) pathway.

**Fig. 2:**
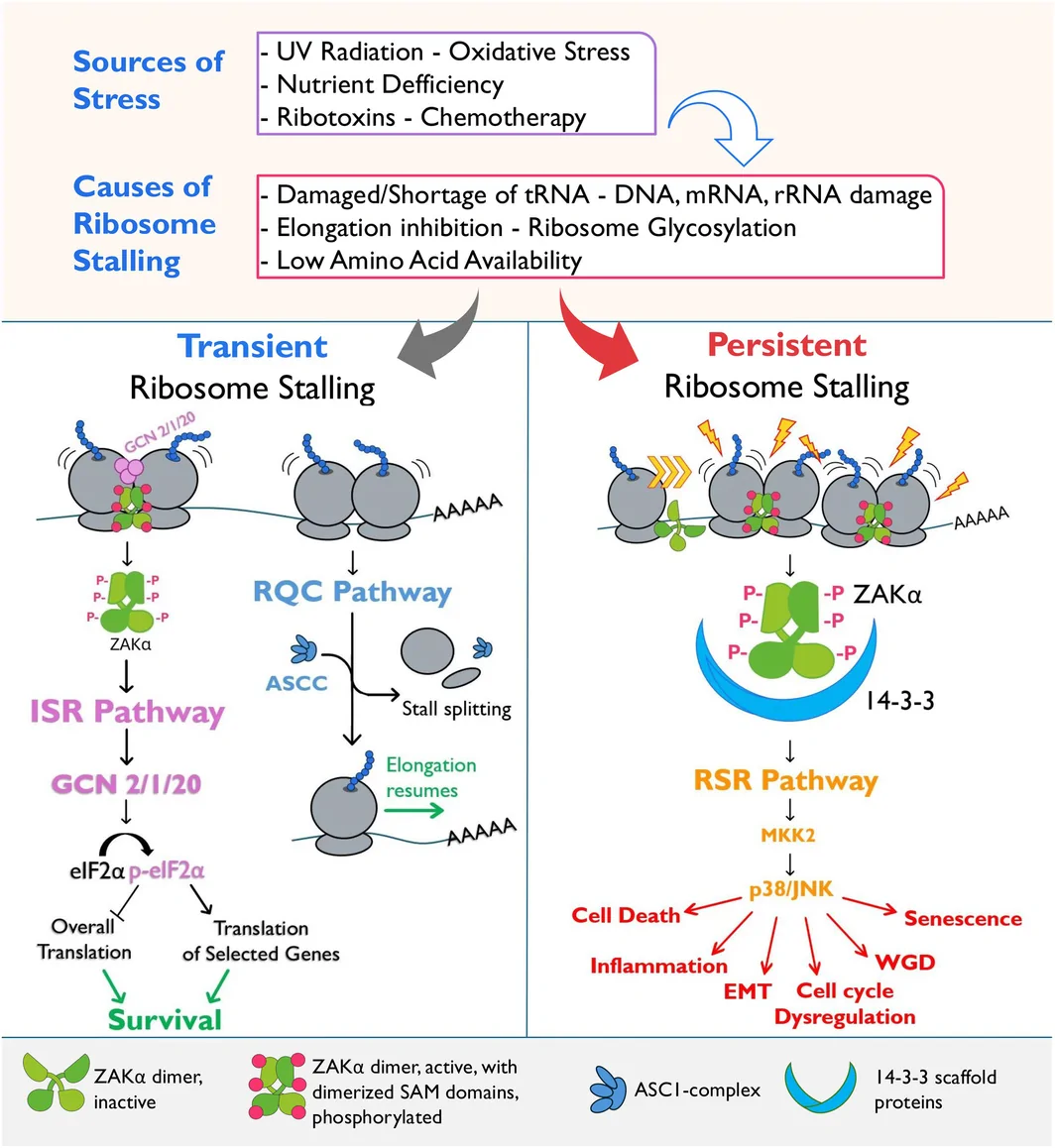
Key mechanisms of stress response to ribosome stalling and collisions. Various external and internal sources of stress may cause ribosome stalling and collisions. When ribosome stalling/collisions are incidental and transient, they can be resolved via integrated stress response (ISR), which involves activation of GCN2/1/20 (general control nonderepressible 2/1/20) and phosphorylation of eIF2α (eukaryotic translation initiation factor 2 alpha). ISR puts a break on total translation and favors translation of stress response genes, supporting cell survival. Transient stalling can also be addressed by ribosome‐associated quality control (RQC) pathway, which results in splitting of stalled ribosomes by ASCC (activating signal cointegrator 1) complex, proteasomal degradation, and resumes normal translation. However, in case of persistent ribosome stalling and frequent collisions, activation of ribotoxic stress response (RSR) via phosphorylation of ZAKα and downstream MKK (MAP kinase kinase) signaling enables a more immediate and radical response resulting in cell death, epithelial–mesenchymal transition (EMT), disrupted cell cycle, WGD, whole‐genome duplication, and other outcomes.

Regulation of translation in cancer also allows for high phenotypic plasticity or ability of cancer cells to rapidly adapt to intrinsic and extrinsic stress caused by dynamic changes in tumor microenvironment, metastasis, and therapeutic interventions [3]. Ribosomes, once considered housekeeping and uniform molecular complexes, are now recognized as dynamic and diverse complexes that play a central role in stress response [8]. Various insults cause ribosome stalling and/or collisions, which trigger three key stress response mechanisms: ribosome‐associated quality control (RQC), the integrated stress response (ISR), and the ribotoxic stress response (RSR) (Figs 1 and 2) [9]. The RQC system responds to stalled translation by removing truncated or abnormal proteins via proteasomal degradation [9]. Meanwhile, the ISR acts as a brake on global protein synthesis but enables selective translation in response to viral infection, amino acid starvation, misfolded protein buildup, and mitochondrial dysfunction [9]. Strikingly, ISR is well‐established as a key mechanism of cancer plasticity [10]. ISR‐mediated selective translation of specialized mRNAs promotes invasiveness, therapy resistance, metabolic adaptation, and survival [10, 11, 12, 13, 14].

The RSR is the most immediate pathway that alerts the cell to translational stress. Like ISR and RQC pathways, RSR occurs when ribosome function is compromised by agents such as translation inhibitors, ribotoxins, chemotherapeutics, or UV radiation [15]. Nonetheless, in contrast to RQC and ISR, RSR induces apoptosis and pyroptosis as opposed to adaptation and survival [15, 16]. Although its mechanism has only been recently understood, RSR‐mediated rapid cell death signaling presents a new therapeutic opportunity. In this review, we summarize the latest understanding of RSR activation, regulation, and RSR‐mediated cellular outcomes relevant to cancer, evaluate current approaches modulating RSR, and discuss their limitations.

## ZAKα—sensor of Ribotoxic stress

Ribotoxic stress usually occurs when translation elongation is inhibited or stalled, leading to ribosome collisions [15]. This disruption can be caused by internal or external stressors that lead to abnormalities or shortages of transfer RNA (tRNA), damage to messenger RNA (mRNA) or ribosomal RNA (rRNA), changes in ribosomal proteins, malfunction of release factors, or direct structural damage to the ribosomes. External triggers of RSR include translation inhibitors (e.g., anisomycin, cycloheximide), ribotoxins (e.g., ricin, Shiga toxin), chemotherapeutic agents (e.g., doxorubicin), and ultraviolet (UV) radiation [17, 18, 19, 20, 21].

Ribotoxic stress is rapidly detected by the MAP kinase kinase kinase ZAKα (also known as MAP3K20 or MLK7). Although RSR was discovered a decade ago, how ZAKα senses ribotoxic stress and triggers stress response was only recently understood [11, 12]. ZAKα protein was believed to detect various conformations associated with abnormal ribosome function [22]. However, two recent studies utilizing biochemistry approach, cryo‐electron microscopy, AlfaFold3 modeling, and RNA crosslinking and immunoprecipitation techniques proposed a model in which ZAKα is highly activated upon collision of two ribosomes or mildly activated at the single stalled ribosomes triggering downstream kinase signaling (Fig. 1) [23, 24]. Based on this model, the ZAKα dimer is autoinhibited via sterile alpha motif (SAM) domains [22, 23, 24]. In homeostasis, inactive ZAKα monomers or dimers ‘sample’ ribosomes by associating with the 40s subunits but only stably bind to collided ribosomes [23]. Ribosome collision or stalling causes SAM domains to dimerize rearranging the autoinhibitory conformation of ZAKα dimer leading to its autophosphorylation (Fig. 1) [23]. Upon activation, ZAKα dissociates from the ribosome, forms a complex with the 14‐3‐3 scaffold protein, and activates the downstream SAPKs p38 and JNK [25].

There must be multiple levels of ZAKα regulation to prevent its premature activation and limit the cytotoxic signaling. However, only a few regulators of ZAKα are known. Vienna L. Huso et al. hypothesized that SERBP1, an RNA‐binding protein that marks ‘dormant’ nontranslating ribosomes, inhibits ZAKα by competing for ribosome binding [23]. ZAKα is also regulated through signal termination via association with β‐TrCP1/2 and subsequent degradation by CRL1 E3 ubiquitin ligase complex [26]. ZAKα protein turnover resets homeostasis and desensitizes cells to prolonged ribotoxic stress. Inhibition of proteasomal degradation of ZAKα, either by ZAKα mutation or bortezomib, maintained sustained activation of ZAKα signaling through JNK and p38 kinases, and increased apoptosis [26]. Mentioned mechanisms of ZAKα regulation were studied in normal cells, but cancer cells may have other mechanisms of inhibiting RSR‐mediated cell death. For instance, we speculate that heightened activation of prosurvival ISR in cancer may prevent RSR activation [10, 26]. Further research of the mechanisms of ZAKα regulation may lead to the discovery of new targets for cancer therapeutics.

Interestingly, ZAKα also activates ISR via one of its kinases, GCN2 [27]. GCN2 has two independent pathways of activation leading to phosphorylation of eIF2A and translation inhibition: free uncharged‐tRNA–dependent pathway and ribosome–dependent pathway. Recently, it was discovered that ribosome collision‐dependent activation of GCN2 depends on ZAKα but not its kinase activity [27]. Hence, ZAKα is the key responder to ribosomal collisions mobilizing the cellular response through both activation of ISR and RSR. Notably, activation of ISR reduces the number of ribosome collisions, prevents further activation of ZAKα, and therefore inhibits RSR [26, 28, 29]. The dual role of ZAKα in both ISR and RSR suggests that in the context of cancer treatment, before pharmacological induction of ribosomal collisions, ZAKα facilitates cancer survival via ISR, but upon strong ribosomal collisions, ZAKα may trigger RSR leading to cell death.

Although little is known about RSR in the cancer context, several studies suggest a model explaining how cell survival or death corresponds to the severity of ribotoxic stress. Ribosomes stalled by translation elongation inhibitors or methylated mRNA are quickly resolved in normal cells by the ASC‐1 complex (ASCC), part of the RQC pathway [30, 31]. In contrast, stalls caused by UV light and 4‐nitroquinoline 1‐oxide resist resolution by ASCC and become persistent and more numerous [30, 31]. Persistent ribosome stalls trigger ZAKα activation, but the downstream outcome depends on the frequency of collisions and ZAKα availability [26]. Under basal conditions, the cell responds to low‐level ribosome collisions by activating p38, inducing G2 arrest, and restraining JNK activity to maintain homeostasis [26]. In this mode, GCN2 activation and mTOR downregulation reduce collisions, while stable ZAKα levels ensure responsiveness to future stress. However, an intermediate level of collisions establishes a tolerance state in which ZAKα degradation by ubiquitination prevents JNK hyperactivity, allowing adaptive gene expression programs to relieve translational stress [26]. Given the high translational capacity of cancer, such a mode can contribute to cancer cells' resilience to frequent ribosome collisions, survival, and therapy resistance [3]. When collisions are very frequent and rapidly accumulating, strong ZAKα‐dependent JNK activation exceeds the apoptotic threshold, and neither GCN2 signaling nor ZAKα degradation can prevent cell death [23, 26]. Identifying cancer‐specific factors that set such an apoptotic threshold can guide therapeutic strategies.

This dose tolerance model underscores the importance of mechanisms that restrain ZAKα activation including GCN2‐mediated ISR signaling, ribosome quality control response (RQC), and activation‐coupled ZAKα proteasomal degradation; potential competitive ribosome binding factors, such as SERBP1, may further modulate this regulation [23, 26, 28]. With this model in mind, a promising next step is to define what sets the apoptotic threshold across different cancers, and to determine whether different cancer types differ in ZAKα abundance, availability, and regulatory control.

## Cellular outcomes of ribotoxic stress response

ZAKα mediates cellular response to ribosomal collisions in consistence with their severity (Fig. 2). When other ribosome control pathways, such as ISR and RQC, fail to resolve severe stalls and collisions, ZAKα activates RSR‐mediated death. Activation of RSR has also been linked to cell cycle regulation, whole‐genome doubling, cell death and inflammation, senescence and aging, and epithelial–mesenchymal transition (EMT) [16, 26, 30, 32]. How these responses correspond to the severity of ribosomal collisions and how the dose tolerance model of RSR contributes to cancer heterogeneity remains poorly understood. The following section provides a more detailed overview of the cellular outcomes triggered by the ribotoxic stress response.

### Apoptosis

Apoptosis serves as a crucial safeguard, allowing damaged or stressed cells to undergo self‐destruction when survival threatens tissue integrity or genomic stability. In cancer, disruption of apoptotic signaling enables damaged or transformed cells to evade elimination, thereby promoting tumor initiation, progression, and resistance to anticancer therapy [33, 34]. Emerging data have shown that ribosome stalling and the ribotoxic stress response are key signaling events that lead to apoptosis. UV radiation is known to cause DNA damage, activating DNA damage response (DDR) pathways and p53‐mediated apoptosis [35, 36]. However, recent studies found that ZAKα‐mediated RSR results in p53‐independent apoptosis in normal and cancer cells [16, 26, 37, 38]. UV radiation causes transcriptome‐wide RNA damage in all cells, resulting in ribosome stalling at damaged codons and ZAK‐mediated JNK activation, whereas DDR occurs only in S‐phase cells [26]. When comparing the roles of RSR and DDR in apoptosis, loss of ZAKα in wild‐type cells nearly abolished apoptosis, whereas p53 deficiency only caused a moderate reduction [26]. These findings indicate that mRNA damage is a key marker of UV‐induced stress, with RSR acting as an earlier trigger of apoptosis independently of p53. Mutations of p53 are found in half of human cancers and genetic alteration or inhibition of p53 is a common mechanism of tumor initiation and progression [39, 40]. The ability of the RSR to induce p53‐independent cell death reframes ribosome stalling from a mere consequence of translational stress to an active signaling event with therapeutic potential for inducing cancer cell death [26, 38]. Interestingly, SLFN11, which is necessary for UAA ribosome stalling and subsequent activation of ZAKα, is inactivated in many chemotherapy‐resistant cancers [38]. Perhaps, to accommodate high translational demands, cancer cells suppress ribosomal stalling and collisions via induction of ISR, SLFN11 inactivation, and possibly other mechanisms, thereby avoiding ZAK‐mediated apoptosis without directly inhibiting ZAKα. Most importantly, the cytotoxic outcomes associated with ZAK‐mediated RSR activation suggest that pharmacological induction of this pathway may represent a promising approach for cancer therapy.

### Inflammation and pyroptosis

Inflammation is a conserved protective response that coordinates immune signaling, tissue repair, and pathogen clearance following injury or stress. Pyroptosis is an inflammatory type of programmed cell death, which canonically depends on caspase‐1 activation via inflammasomes. Several studies have reported that ZAKα directly phosphorylates NLRP1, thereby promoting NLRP1 inflammasome assembly and pyroptosis in human keratinocytes [41, 42, 43]. Notably, NLRP1 expression was a key determinant of cell fate after UV radiation [26]. NLRP1 was necessary for the induction of ZAKα‐mediated pyroptosis in keratinocytes, whereas in cells lacking NLRP1, UVB‐induced damage triggered ZAKα‐ and JNK‐mediated apoptosis [26]. Similarly, prolonged ribotoxic stress activates pyrin‐dependent inflammasome, leading to secretion of proinflammatory cytokines such as IL‐1beta and IL‐18, and pyroptosis in 293T cells [44].

Presented evidence suggests a strong role of RSR in pyroptosis in normal skin cells but further investigation needs to be done using cancer models. In the context of cancer, abnormal or hyperactivated inflammatory signaling can promote tumor initiation and progression by altering the tumor microenvironment and aiding cancer immune evasion [45]. However, pyroptosis can serve as a viable method of killing cancer cells when they are resistant to apoptosis [46].

### Cell cycle and whole‐genome doubling

Proper cell cycle regulation ensures that cells only progress through division when essential processes such a DNA replication and protein synthesis are intact. In response to cellular stress, cell cycle arrest acts as a protective checkpoint, allowing cells to pause proliferation and restore homeostasis before committing to survival or death pathways. RSR and ZAKα regulate key cell cycle transitions via p38 signaling [47].

As mentioned in the previous section, distinct types of ribosome stalls differ in their sensitivity to ASCC‐mediated ribosome‐associated quality control, which promotes the disassembly of stalled ribosomes [30]. ASCC‐resistant stalls led to prolonged cell cycle arrest in G2 phase via ZAKα‐mediated p38, while less persistent stalls caused only transient G2 arrest [30, 31].

Although prolonged G2/M cell cycle arrest can result in apoptosis and is an existing therapeutic strategy, G2 arrest can allow cancer cells to repair DNA damage and result in treatment resistance. Moreover, loss of cell cycle control can lead to whole‐genome doubling (WGD) and aneuploidy, characteristics often associated with tumor metastasis and poor prognosis. McKenney et al. showed that ribotoxic stress caused endoreplication through ZAKα and p38 signaling in a dose‐dependent manner through inhibition of CDK1 and CDK4/6 [32]. Interestingly, this study used a ribotoxic stress model similar to that employed by Stoneley et al. in the earlier study [30]. Both studies treated MCF10A cells with anisomycin; however, the study reporting the G2 exit followed by WGD used a higher concentration and longer exposure to anisomycin than the one observing sustained G2 arrest [30, 32]. Considering both studies and the dose tolerance model of ZAKα‐mediated stress responses, the therapeutic induction of RSR to promote cancer cell death may require strategies beyond simply increasing the intensity of ribotoxic stress, as excessive stress may instead trigger undesirable outcomes such as endoreplication. Additionally, SAPKs are known to influence senescence and aging, so ZAKα‐mediated WGD may be another factor contributing to aging‐related genetic instability and tumor development.

### Senescence and aging

Cellular senescence is a permanent state of cell cycle arrest that accumulates in response to stress and damage, while aging involves the gradual decline in cellular homeostasis over time. Both processes are closely connected to defects in proteostasis and translational control, which make cells more vulnerable to stress. A study in *C. elegans* and *S. cerevisiae* demonstrated that ribosome stalling increases during aging and is associated with impaired clearance of ribosome‐associated quality control (RQC) substrates [48].

Consistent with these observations, a study using mammalian cell culture and mouse models showed that aging decreases METTL1 expression, reducing N7‐methylguanosine (m7G46) modification of tRNA species and triggering rapid tRNA decay (RTD) [49]. Consequently, METTL1 deficiency increased ribosome stalling, activating both ISR and RSR. Notably, ISR and RSR signaling, mediated by GCN2, ZAKα, p38, and JNK, led to an increase in senescence‐associated secretory phenotype (SASP) factors, such as IL‐6, CXCL8, and IL‐1β [49]. These findings provide direct evidence that activation of ISR and RSR contributes to the establishment of cellular senescence. Additionally, combining three inhibitors of GCN2, p38, and JNK proved effective against senescence in METTL1 knockdown IMR90 cells and Mettl1‐deficient mice [49]. These findings raise the possibility that aging shifts the cellular response to persistent ribosome stalling from apoptosis toward senescence. However, the mechanisms that increase cellular tolerance to persistent ribotoxic stress during aging remain poorly understood and warrant further investigation.

The relationship between RSR‐induced senescence and cancer is likely to be complex. Although senescence suppresses the proliferation of damaged cells, the accompanying SASP can promote tumor progression through chronic inflammation, extracellular matrix remodeling, and immune modulation [50]. In addition, senescent cancer cells are resistant to apoptosis by expressing anti‐apoptotic proteins maintaining their survival [50, 51]. Therefore, therapeutic strategies aimed at activating RSR should consider the balance between apoptosis and senescence. Inducing sublethal ribotoxic stress may unintentionally promote a protumorigenic senescent phenotype instead of eliminating cancer cells. Understanding the determinants that govern this cell fate decision will therefore be important for the therapeutic development of RSR‐based approaches.

### Epithelial–mesenchymal transition and cancer metastasis

Epithelial–mesenchymal transition (EMT) is a dynamic cellular process in which epithelial cells lose polarity and cell–cell adhesion while gaining mesenchymal traits that increase motility and invasiveness. In cancer, abnormal activation of EMT promotes tumor metastasis and resistance to environmental stress and therapies. ZAKα has been shown to play a role in EMT and tumor metastasis. The authors demonstrated that knocking down ZAKα in a triple‐negative breast cancer cell line (MDA‐MB‐231) reversed its mesenchymal phenotype and reduced cell migration [52]. Additionally, ZAK‐deficient MDA‐MB‐231 cells injected into immunodeficient mice showed decreased bone metastasis compared to wild‐type cells. Mechanistically, ZAKα knockdown resulted in downregulation of the EMT transcription factor ZEB1 and a shift in CD44 splicing from a mesenchymal to an epithelial isoform [52]. However, it is possible that the authors did not fully consider ISR signaling mediated by ZAK‐dependent GCN2 activation. We suggest that the observations reported by Li et al. may be explained by a ‘low‐collision’ context, in which GCN2‐dependent ISR signaling promotes cancer cell survival and EMT rather than apoptosis [52]. Given the established role of the ISR in EMT, further investigation is needed to determine whether RSR‐associated SAPK signaling similarly contributes to EMT and metastatic progression [11]. Such studies will be important for evaluating the safety and therapeutic potential of strategies aimed at inducing RSR in cancer cells.

## Evaluating current approaches and limitations of RSR‐modulating agents in cancer therapeutics

Modulating cellular stress pathways as a therapeutic strategy presents several unique challenges. A danger inherent to altering any one of these pathways is unintended downstream effects that either modify resistance to existing therapies or promote secondary carcinomas. In the case of the ZAKα‐mediated ribotoxic stress response, activating this pathway has the advantage of being able to promote apoptosis in cells, but the same pathway has also been shown to promote inflammation and the epithelial–mesenchymal transition, both major contributors to cancer pathology. Combination treatment with agents that mitigate the RSR may therefore be used to reduce side effects.

In this section, we evaluate the effectiveness and limitations of existing therapeutics, which either induce or inhibit RSR. In doing so, we once underline the importance of understanding this pathway in an oncogenic context, as altering its expression can drastically affect the progression of cancer.

### FDA‐approved RSR‐inducing agents

Several FDA‐approved chemotherapeutic agents induce the RSR as the primary means of opposing cancer growth (Table 1). One such agent is the plant alkaloid homoharringtonine (HHT), also known as omacetaxine mepesuccinate, FDA‐approved for use in chronic myeloid leukemia. HHT binds and inhibits the ribosomal A‐site to inhibit protein synthesis and translation, inducing apoptosis through the p38/JNK‐mediated ribotoxic stress response. Its effectiveness in solid tumors has also been demonstrated in triple‐negative breast cancer cells; however, it also promotes the deubiquitination and nucleolar accumulation of EMT‐modulating transcription factor Snail1 in solid tumors, heightening resistance to HHT [53, 54, 55]. Another RSR‐inducing agent demonstrated to combat liquid tumor growth is arsenic trioxide (ATO), an arsenic compound used to treat acute promyelocytic leukemia (APL). ATO induces ribosome stalling and RSR‐driven apoptosis of cancer cells by binding nascent polypeptides, disrupting protein synthesis. A study conducted by Xie et al. demonstrates that ATO, paired with an inhibitor of p97 (a ribosome‐associated ATPase, which facilitates the degradation of misfolded proteins via the ERAD pathway), induces ZAKα/p‐JNK‐mediated apoptosis in both APL and non‐APL AML cell lines [56]. Further study is required to establish ATO as a viable RSR‐inducing chemotherapeutic for solid malignancies, as well as addressing severe side effects caused by its toxicity [57]. The antibiotic doxorubicin (DOX) is used in the treatment of various cancers, including but not limited to those of the breast, bladder, thyroid, and ovary. It induces translational stress by acting as an intercalator, inserting between DNA base pairs to inhibit protein synthesis, as well as promoting reactive oxygen species (ROS) accumulation and inhibiting topoisomerase II activity. DOX has been demonstrated to induce apoptosis through ZAKα‐mediated RSR in osteosarcoma cells and keratinocytes *in vitro*, with reduced ZAKα expression also reducing the effectiveness of DOX [58, 59].

**Table 1:** Summary table of RSR‐modulating agents and their effect on cancer.

Cancer type(s)	Agent	Model	Mechanism (↓ ↑)	Outcome	Limitations	Status	References
Breast	Omacetaxine mepesuccinate / Homoharringtonine (HHT)	*In vitro*: MDA‐MB‐231, MDA‐MB‐468, MDA‐MB‐157, CAL‐51 *In vivo*: Swiss nude mice with MDA‐MB‐231 or MDA‐MB‐468 xenografting	↓Cell viability G1/S cell cycle arrest ↓Mcl‐1 protein	Apoptosis (cells), reduced tumor volume (mice)	Quick degradation in patients, some toxic side effects	FDA‐approved (2012)	[54]
Breast CML Lung (NSCLC) AML	Omacetaxine mepesuccinate / Homoharringtonine (HHT)	*In vitro*: HCC1806, K‐562, HEK‐293, Hs 578T, SUM159, NCI‐H1299, HEK‐293FT, A549, OCI‐AML2, MOLM‐13; clinical breast tumor samples *In vivo*: BALB/c nude mice with A549 xenografting	↑p‐JNK ↑USP36 and Snail1 expression ↑Snail1 nucleolar localization	Very slight induction of apoptosis. Increased ribosome biogenesis	Minimal apoptosis unless paired with USP36 and/or Snail1 knockdown.	FDA‐approved (2012)	[55]
APL AML Histiocytic lymphoma APL	Arsenic trioxide (ATO)	*In vitro*: NB4, MV4‐11, NOMO‐1, MOLM‐13, THP‐1, U937, HL‐60, Kasumi‐1 *In vivo*: NSG mice with Kasumi‐1 xenografts	↑ROS ↑GCN2‐ATF4 activity ↑p‐ZAKa ↑p‐JNK	Apoptosis (cells), reduced tumor growth (in mice; synergistic with NMS873)	Effectiveness limited to APL.	FDA‐approved (2000)	[56]
Bone (osteosarcoma)	Doxorubicin	*In vitro*: HOS (CRL‐1543)	↑p‐ZAKa ↑Caspase‐3 cleavage ↓Bcl‐xL	Apoptosis	Risk of cardiotoxicity	FDA‐approved (1974)	[11]
Skin (SCC)	Sorafenib	*In vitro*: HaCaT and NHEK; squamous cell carcinoma biopsy samples *In vivo*: Hairless mice	↓p‐JNK ↓Caspase‐3 cleavage	Reduced doxorubicin‐ induced apoptosis, increased tumor development in mice	Worsens cancer progression	FDA‐approved (2005)	[61]
Skin (SCC)	Vemurafenib/PLX4720	*In vitro*: SRB1, SRB12, COLO16, HaCaT, WM35, A375	↓p‐JNK ↓p‐p38	Reduced doxorubicin‐ induced apoptosis	Could potentially promote cancer growth in patients treated with vemurafenib	FDA‐approved (2011)	[64]
Lung (NSCLC)	Exotoxin A (EXOA)	*In vitro*: A549 Primary human corneal (pHCECs) Primary nasal epithelial cells (pHNECs)	↑NLRP1 inflammasome complex activity ↑p‐JNK ↑p‐p38	Apoptosis, pyroptosis, inflammation, epithelial barrier disruption		FDA‐approved (2018)* *In the form of immunotoxins, which were not used in this study	[66]
Bone (osteosarcoma)	Emetine	*In vitro*: U2OS	↑p‐p38	Very slight induction of the RSR	High risk of toxicity	Approved pre‐FDA	[22]
Lung (NSCLC)	Emetine	*In vitro*: A549, H1299	↑p‐p38 ↑p‐JNK	Inhibited cancer cells' invasion and migration	High risk of toxicity	Approved pre‐FDA	[68]
Bone (osteosarcoma)	Ricin	*In vitro*: U2OS	↑p‐p38 ↑p‐JNK	Rapid and strong induction of the RSR	High risk of toxicity	Not FDA‐approved	[22]
Colorectal adenocarcinoma	Shiga toxin	*In vitro*: HCT‐8	↑p‐JNK ↑Caspase‐3 cleavage	Induction of RSR and apoptosis in human colon adenocarcinoma cells.	High risk of toxicity	Not FDA‐approved	[76]
Prostate	Anisomycin (ANS)	*In vitro*: DU145	↑p‐JNK ↑Caspase‐3 cleavage	Increased sensitivity to Fas‐mediated apoptosis	Risk of toxicity at higher doses	Not FDA‐approved	[81]
Lung (NSCLC)	2′,5′‐oligoadenylate synthase (2‐5A)	*In vitro*: A549, mouse bone marrow macrophages	↑ RNase L ↑ ZAKα ↑p‐JNK ↑p‐p38 ↑p‐p38 ↑PARP cleavage	Activation of RSR‐mediated apoptosis	2‐5A is quickly degraded by cellular enzymes.	Not FDA‐approved	[21]
T‐ALL Breast Cervical Soft tissue (rhabdomyosarcoma, fibrosarcoma, Ewing sarcoma) Liver Skin (melanoma) Prostate Kidney (RCC) Colon Glia	Portimine A toxin	*In vitro*: Jurkat, HCC1806, HeLa, MC38, Lenti‐X™ 293T, RD, HT‐1080, A673, MCF‐7, MDA‐MB‐231, HepG2, B16‐F10, SUM159, LNCaP, 786‐O, 4T1, GBM‐A, GBM‐F, LN229, U87EGFRvIII, U118 *In vivo*: C57BL/6 mice	↑Cleaved caspase‐3 ↑Cleaved PARP1	Activation of RSR‐mediated apoptosis	Apoptotic activity of portimine A is dependent on its ability to target and bind NMD3	Not FDA‐approved	[89]
Breast	Anisoplin	*In vitro*: MCF‐7, HEK293	↑p‐JNK ↓NFκB	Activation of RSR‐mediated apoptosis	High risk of toxicity	Not FDA‐approved	[90]
Colorectal adenocarcinoma	DHP‐2 (7‐[3‐fluoro‐4‐aminophenyl‐(4‐(2‐pyridin‐2‐yl‐5,6‐dihydro‐4H‐pyrrolo[1,2‐b]pyrazol‐3‐yl))]‐quinoline)	*In vitro*: Hct‐8, Vero, intestinal epithelial cells	↓phospho‐c‐Jun ↓p‐ATF2 ↓Caspase‐3 cleavage ↓IL‐8 ↓p‐JNK ↓p‐p38	Activation of RSR‐mediated apoptosis		Not FDA‐approved	[19]
Lung (NSCLC) Breast	DHP‐2 (7‐[3‐fluoro‐4‐aminophenyl‐(4‐(2‐pyridin‐2‐yl‐5,6‐dihydro‐4H‐pyrrolo[1,2‐b]pyrazol‐3‐yl))]‐quinoline)	*In vitro*: A549, HEK293T, MDA‐231‐1833	↓p‐p38 ↓pSMAD2 ↓pSMAD3	Reduced TGF‐β signaling and TGF‐β‐mediated cell invasion		Not FDA‐approved	[21]

The increase of ROS caused by doxorubicin is a major contributor to its cardiotoxic side effects. A study conducted by Zhou et al. suggested the cardiotoxicity associated with doxorubicin treatment was reduced in rats also treated with Bcr‐Abl tyrosine kinase inhibitor nilotinib [60]. The authors postulate that nilotinib's function as an off‐target ZAKα inhibitor could be significant to this finding, suggesting RSR signaling could play a role in the cardiotoxicity and inflammation experienced by patients undergoing DOX treatment, but further investigation is required to solidify the connection between ZAKα and cardiotoxicity [60].

Chemotherapeutic agents that directly activate the ribotoxic stress response in cancer cells greatly enhance pro‐apoptotic outcomes, both as monotherapies and when used in combination treatments (Table 1). However, more research is required in order to determine what harmful side effects may result from prolonged activation of the pathway. Further investigation may also indicate how effective modulation of the RSR is in liquid versus solid tumors, as homoharringtonine and arsenic trioxide are primarily used to treat leukemias, while doxorubicin is effective across a much broader range of cancers.

### RSR inhibition limits cancer cell death

Several FDA‐approved chemotherapeutic agents have also been observed to inhibit the activation of the RSR through off‐target inhibition of ZAKα (Table 1). These include sorafenib, nilotinib, and ponatinib, receptor tyrosine kinase inhibitors, which limit RSR activation through off‐target inhibition of ZAKα. A study conducted by Wong et al. showed that administration of these drugs alongside doxorubicin (DOX) reduced activation of ZAKα and p‐JNK by DOX, lessening its proinflammatory side effects [61, 62]. Pairing drugs that cause the RSR with a ZAK inhibitor may limit some of the RSR's less desirable outcomes in cancer patients.

The BRAF V600 inhibitor vemurafenib, which targets mutant BRAF in malignant melanomas, has also been demonstrated to inhibit the RSR through off‐target binding to ZAKα [63, 64]. Vin et al. postulate this subsequent suppression of p‐JNK‐mediated apoptosis contributes to the development of secondary malignancies associated with BRAF inhibitors [64]. The results of this study also indicate inhibition of ZAKα directly reduces RSR‐induced cancer cell apoptosis.

Laboratory reagents used to inhibit the ZAKα‐driven ribotoxic stress response may also demonstrate potential usage in cancer therapeutics. Namely, DHP‐2 (7‐[3‐fluoro‐4‐aminophenyl‐(4‐(2‐pyridin‐2‐yl‐5,6‐dihydro‐4H‐pyrrolo[1,2‐b]pyrazol‐3‐yl))]‐quinoline), a ZAKα‐specific inhibitor, significantly abrogates some of the harmful side effects of RSR activation. DHP‐2 counters the proinflammatory and pro‐apoptotic effects of the RSR induced by Shiga toxins and ricin in Hct‐8 adenocarcinoma cells [19]. DHP‐2 also reduces ZAKα‐mediated TGFβ signaling and cancer cell invasion in breast and lung cancer cell lines [65]. DHP‐2 could potentially be used in combination with RSR‐inducing therapeutic agents to limit harmful and/or pro‐oncogenic outcomes.

Collectively, these findings highlight the context‐dependent implications of ZAKα inhibition in cancer therapy. While inhibition of ZAKα may mitigate undesirable consequences of RSR activation, including excessive inflammation and potentially pro‐oncogenic signaling, it may also attenuate RSR‐mediated apoptosis and thereby compromise the efficacy of therapies that rely on ribotoxic stress to eliminate cancer cells. Thus, combining RSR‐inducing agents with ZAKα inhibitors may require careful optimization to suppress harmful downstream effects without undermining the desired cytotoxic response. Further investigation is also needed to determine whether off‐target ZAKα inhibition by existing anticancer drugs contributes to therapeutic resistance by enabling cancer cells to evade RSR‐mediated apoptosis under conditions of translational stress.

### Novel RSR‐inducing agents

Induction of the ribotoxic stress response has shown itself to be a highly viable anti‐oncogenic therapeutic strategy, both on its own and in combination with other therapeutics. This has provided a strong incentive for novel and existing RSR‐modulating agents to be evaluated in the context of ameliorating cancer. This section discusses non‐FDA‐approved agents, which have been demonstrated to induce the RSR in cancer cells in laboratory settings, as well as FDA‐approved agents, which induce the RSR but are not yet implemented in the context of cancer and require further investigation.

A strong candidate for future therapies is immunotoxins developed from ribotoxins. Ribotoxins secreted by fungi and bacteria cleave the phosphodiester bond in the backbone of rRNA to trigger various cellular stress responses and cell death. This includes the ribotoxic stress response, as seen in studies of ribotoxins diphtheria toxin (DT) and exotoxin A (exoA), shown to induce the RSR to drive apoptosis and pyroptosis *in vitro* [43, 66]. To take advantage of these agents without endangering wild‐type cells, such toxins can be developed into recombinant immunotoxins, chimeric molecules consisting of an antibody targeting a cancer cell marker and a toxin, which halts protein synthesis and induces apoptosis. Both DT and exoA have been used to develop several FDA‐approved immunotoxins: from DT, tagraxofusp (Elzonris) and denileukin diftitox (Ontak); from exoA, moxetumomab pasudotox (Lumoxiti). Given that the original toxins induce apoptosis through the RSR, it is likely immunotoxins based on them use a similar mechanism, though further study is required to clarify their precise mechanism of action.

The ipecac‐derived drug emetine is FDA‐approved for the treatment of amoebiasis and induces translational stalling, ribosome collisions, and the RSR by binding the 40S ribosomal subunit [67, 68, 69]. Although emetine has been demonstrated to activate the RSR in cancer cells *in vitro*, it does so weakly when compared to other agents, such as anisomycin and ricin [22]. However, given that prolonged RSR activation may contribute to inflammation, weak activation of the pathway may be preferable. Recent studies have also shown emetine to be effective in reducing both solid cancer and leukemic growth [67, 68, 69]. Further investigation into emetine as a novel cancer inhibitor may demonstrate the potential for RSR activation in cancer therapy.

The potent plant toxin ricin, which induces ribotoxic stress by cleaving the sarcin‐ricin loop (SRL) in rRNA, has been used to develop several immunotoxins utilizing its cytotoxic A‐chain [19, 70, 71]. These immunotoxins have proven effective in melanoma and breast cancer cell lines, as well as in a number of clinical trials [72, 73, 74]. However, the propensity of ricin‐based immunotoxins to cause vascular leak syndrome has thus far limited their therapeutic use [75]. Another type of proposed immunotoxin‐based therapies is based on bacterial Shiga toxins, which cleave a single adenine residue within the 28s subunit of the ribosome to induce apoptosis and inflammation via ZAK‐mediated RSR [76, 77, 78]. Although not yet FDA‐approved, immunotoxins developed from Shiga toxin type 2 (Stx2) have been shown to successfully induce apoptosis in HT‐29 colon cancer cells *in vitro* [79].

A number of RSR‐inducing agents not yet FDA‐approved for clinical use have also shown some therapeutic potential. One such agent is the translation inhibitor anisomycin (ANS), which binds the 60S ribosomal subunit to quickly induce ribosomal collisions, translational stalling, and apoptosis. At low concentrations, anisomycin decreases the viability and proliferation of mouse melanoma cells and promotes senescence [80]. ANS has also been studied in combination with other antitumorigenic agents, where it has been found to sensitize cancer cells to the other drugs, increasing rates of apoptosis relative to treatments without ANS [81, 82]. This sensitivity may be due to ANS‐induced differential regulation of several immunomodulatory genes, upregulating CD58 and ICAM4 while downregulating MHC‐I, increasing the cells' vulnerability to cytotoxic NK cells [83]. ANS has not been approved for clinical trials, and studies in model systems have primarily focused on its use as an antineurodegenerative agent. Anisomycin has not seen approval for clinical trials, and its ribotoxicity at high doses has been demonstrated in animal models, proving to be a challenge for future therapeutic consideration [84].

2′‐5′‐oligoadenylate synthase (OAS), also known as 2‐5A, is an antiviral enzyme that, upon detecting viral dsRNA, synthesizes 2′‐5′‐oligoadenylates. These molecules then bind to and activate the endoribonuclease RNase L, which cleaves ssRNAs and inhibits protein synthesis. This process activates the effectors of the ribotoxic stress response, namely ZAKα, JNK, and p38, which induce proinflammatory signaling and apoptosis in human lung cancer cells [21]. OAS enzymes are also highly upregulated in a number of cancer types and have also been linked to both prosurvival and pro‐apoptotic functions in the tumor microenvironment [85, 86, 87]. Further research is needed to explore their therapeutic potential, whether as a target for inhibition or activation.

The cyclic imine toxin Portimine A is produced by the marine dinoflagellate *Vulcanodinium rugosum*. By binding 60S assembly protein NMD3, Portimine A induces translational stalling and triggers the ribotoxic stress response via ZAKα and p38, as well as the formation of the NLRP1 inflammasome, thereby driving inflammation and apoptosis [42, 88]. Recent studies have demonstrated that it is able to inhibit translation through its interactions with NMD3, the 60S ribosomal export protein, though the precise mechanism is not yet known. When assessed against cancer cell lines rather than human primary cells, treatment showed acute toxicity in cancer cells but not in normal cells, indicating some degree of selectivity for inducing apoptosis in cancer cells [89].

Anisoplin is a fungal ribotoxin that, like ricin, cleaves a single phosphodiester bond within the SRL of rRNA to induce translational stalling and ribotoxic stress [90]. Published findings by Patra et al. in 2024 entailed the apoptosis of MCF‐7 breast cancer cells by recombinant anisoplin, highlighting another ribotoxin for potential future development into an effective immunotoxin [90].

Many of the agents discussed in this section display potential for RSR‐induced tumor shrinkage and cancer cell apoptosis, though a better understanding of their long‐term side effects is required prior to their clinical application. Ribotoxins, while capable of reliably inducing the RSR and apoptosis in a wide range of solid cancer cell lines, present major health risks to the body. Yet, using these toxins to engineer immunotoxins appears to be the safest means of using them for cancer therapy.

### Limitations

Therapies that induce apoptosis via the ZAKα‐mediated ribotoxic stress response do so by inducing translational stalling and ribosome collisions. However, ZAKα activation will occur only when the stress has reached a certain threshold; mild stress will more likely result in activation of pro‐survival ISR signaling via GCN2 [26]. Thus, for therapies that induce the RSR, determining precise apoptotic thresholds presents a major challenge, as failure to reach said threshold could have adverse, even pro‐oncogenic outcomes. In addition, Sinha et al. observed how degradation of ZAKα in cells undergoing moderate ribotoxic stress serves to desensitize them to future stressors, which could limit the effectiveness of long‐term treatments [26].

Several approaches could be taken to circumvent these methods. Rather than targeting ribosome components, future studies could investigate a chemical means of binding ZAKα directly to cause SAM domain dimerization and activation independent of ribosome collisions. Another potential approach would be the development and delivery of a mutant hyperactive ZAKα, although this could have a number of harmful side effects and would likely require administration of a ZAKα inhibitor in order to protect healthy tissue. Thus far, ribotoxin‐based immunotoxins show the highest potential for future RSR‐based therapies, as the antibody fragment bound to the toxin allows for specific targeting of cancer cells. Furthermore, additional investigation into the role of ZAKα across different cancer types and tumor microenvironments may indicate which cancers show the best response to RSR‐mediated apoptosis, as well as which are susceptible to less beneficial outcomes, such as EMT and secondary malignancies.

## Conclusion

The studies reviewed here collectively identify the RSR as a central signaling hub that converts translational stress accompanied by ribosomal stalling and collisions into cellular outcomes that can be either adaptive or deadly to the cell, including apoptosis, inflammation, and pyroptosis, cell cycle arrest, whole‐genome doubling, senescence, and epithelial–mesenchymal transition (EMT). At its core, ZAKα functions as a sensor kinase that detects ribosome collisions/stalling and coordinates ribotoxic stress response through downstream stress‐activated protein kinase (SAPK) signaling. Its activation is tightly regulated by multiple layers of control, enabling cellular responses that scale with the severity of ribotoxic stress. However, the regulatory network keeping ZAKα activation in check requires further investigation to shed light on cancer‐specific or tissue‐specific differences and guide better therapeutic strategies.

In cancer, this RSR axis presents a therapeutic promise that needs to be addressed with caution. Strong RSR activation can bypass typical survival mechanisms such as p53 deficiency, using ribosome‐targeting agents to induce tumor cell death. However, dysregulated or context‐dependent RSR signaling, often heightened by oncogenic stress, may promote genomic instability, proinflammatory tumor microenvironment, and EMT‐related metastasis, highlighting its dual nature.

Critically, RSR does not operate in isolation but intersects with other pathways like the integrated stress response (ISR), where GCN2 detects uncharged tRNA species to suppress translation and trigger eIF2α phosphorylation. GCN2 plays a crucial role in cell survival and proliferation under nutrient‐deficient conditions and importantly prevents further ZAKα activation by reducing ribosome collisions. Inhibition of GCN2 was already suggested as a therapeutic strategy to tackle chemoresistance in TP53‐WT quiescent tumor cells and sensitize resistant tumor cells to RNA PolI inhibitors [54]. However, it is possible that inhibition of GCN2 may lead to activation of ZAKα, resulting in either desirable cancer cell death, or compensation for the lack of GCN2 activity by promoting cancer survival, metastasis, or senescence. Moreover, an inhibitor of GCN2, GCN2iB, was found to directly inhibit ZAKα, reducing the cytotoxicity [91]. Several GCN2 inhibitors have a similar chemical structure to ZAKα inhibitors [91]. Such unintended inhibition of ZAKα may reduce the effectiveness of GCN2 inhibitors, but combination with ZAKα activators may solve this issue. Furthermore, the overlapping functions of ZAKα in mediating both the ISR and RSR should be considered in experimental design and data interpretation. We suggest that, to minimize the contribution of ISR signaling when studying ZAKα in the context of RSR activation, experimental models should include conditions that generate high‐frequency ribosomal collisions characteristic of ribotoxic stress.

Recent advances suggest that the RSR functions within a broader, highly context‐dependent stress signaling network that is critically relevant to cancer biology. Future research must clearly define how the ISR, driven by GCN2‐mediated phosphorylation of eIF2α, activates ATF4 to determine cell fate under stress by balancing adaptive survival and apoptotic outcomes. Central to this coordination is ZAKα, which links RSR signaling through JNK/p38 MAPK pathways with ISR activation via GCN2, thereby integrating ribotoxic and translational stress signals [26, 27, 28, 29].

Notably, cancer cells rely on elevated ribosome biogenesis to sustain rapid proliferation, a process driven by oncogenes such as MYC, RAS, and PI3K [92, 93]. This hyperactivation of ribosome production increases their susceptibility to ZAK‐mediated apoptotic signaling. Understanding these interconnected pathways will be essential for positioning translational addiction and ribotoxic stress as exploitable cancer vulnerabilities. Such insights could enable the development of biomarker‐guided therapeutic strategies that specifically target cancer cell dependencies on dysregulated ribosome biogenesis and stress adaptation mechanisms.

Future work should also further investigate the relationship between RSR and cancer survival and therapy resistance. For instance, the study by Park et al. defined that induction of ribosomal collisions can act as a switch turning off the tumor resistance and enabling RSR‐mediated cell death [37]. Hence, future research should evaluate the effectiveness of combination therapy with inducers of ribosome collisions or RSR in therapy‐resistant tumors.

Techniques evaluating protein synthesis such as ribosome profiling, single cell phosphoproteomics, spatial multi‐omics, and emerging techniques such as Ribo‐tweezers will reveal dynamic interactions between ZAKα, its regulators, and other stress response pathways *in vitro* and *in vivo*, guiding precision strategies that utilize RSR anticancer potential while avoiding its adverse effects [94, 95]. Understanding mechanisms that restrain ZAKα activation and define cellular tolerance to the severity of ribosome collisions in the context of cancer can uncover new therapeutic targets and suggest better combination treatments in the future. In our opinion, decoding this intricate network of stress response pathways will redefine translational stress as a fundamental aspect of cancer plasticity and vulnerability.

## Conflict of interest

The authors declare no potential conflicts of interest. The content is solely the authors' responsibility and does not necessarily represent the official views of the National Institutes of Health.

## Author contributions

AK, MA, MS, ET, AR, SS, AS, and CP were involved in writing, review and editing. AK and ET were involved in visualization. All authors have read and agreed to the published version of the manuscript.
